# Anaesthetic Management in Mucopolysaccharidoses Patients: Clinical Experience in a Tertiary Hospital

**DOI:** 10.7759/cureus.27474

**Published:** 2022-07-30

**Authors:** Andreia Machado, Diana Rodrigues, Amélia Ferreira, José Dias, Patrícia Santos

**Affiliations:** 1 Anaesthesiology, Centro Hospitalar e Universitário de São João, Porto, PRT; 2 Anesthesiology, Centro Hospitalar e Universitário de São João, Porto, PRT; 3 Department of Surgery, Anesthesiology and Intensive Care Unit, Faculty of Medicine of University of Porto, Porto, PRT

**Keywords:** airway management, perioperative care, anaesthesia, paediatrics, mucopolysaccharidoses

## Abstract

Introduction: Mucopolysaccharidoses (MPS) are a group of rare inherited metabolic diseases caused by the deficiency of enzymes required to degrade glycosaminoglycans (GAGs) in the lysosome with progressive and multiorgan involvement. Due to its progressive nature, a large number of patients need surgical intervention, which is associated with higher perioperative morbidity.

Methods: We retrospectively reviewed 29 medical records regarding the peri and post-operative anaesthetic care of 10 children with mucopolysaccharidoses at a tertiary hospital in Northern Portugal between 2010 and 2021. A comprehensive review of the literature is also included.

Results: Twenty-nine medical records of 10 patients with a mean age of 8.8 years old were included. At the time of the procedure, 65.5% of patients were under enzymatic replacement and all had some type of organ involvement.Each patient was subjected to 2.9 elective procedures on average, with the lowest age of two years at the first procedure. Regarding surgical specialties, 41.4% of interventions were orthopaedic. Balanced anaesthesia with inhalational induction and spontaneous breathing was performed in 48.3% of cases, with no reports of difficult bag-mask ventilation. Endotracheal intubation in the operating room was performed in nine cases. Direct laryngoscopy with a Macintosh blade was the first option in seven patients. Success was achieved only in three cases. Failed intubation was managed with a paediatric C-MAC® video laryngoscope (VLG; one case) and fiberoptic bronchoscopy (two cases). Video laryngoscopy and fibroscopy were the first choices in one case each. Post-operative complications were registered in 10 procedures (34.5%), with airway and pulmonary complications in 17.2%. All complications occurred in different patients except for post-operative nausea and vomiting, which were registered in three cases, two of them in the same patient at different orthopaedic surgical interventions.

Discussion: The high percentage of direct laryngoscopy could be related to the greater number of interventions performed in a period when indirect methods were not so widely used in anaesthetic practice, and the high rate of failure highlights the importance of transitioning to indirect techniques upon the first attempt. The high percentage of patients under enzymatic treatment can explain the low number of adverse airway events. High-quality evidence regarding MPS is lacking in the literature. Prospective studies would improve the perioperative care of this particular subset of patients.

Conclusion: Patients with MPS represent a huge challenge for the anaesthesiologist, mainly in view of airway management. The integration of all available data in clinical practice is essential to optimize perioperative management of mucopolysaccharidoses patients’ management and improve clinical outcomes.

## Introduction

Mucopolysaccharidoses (MPS) are a group of inherited metabolic diseases caused by the deficiency of enzymes required to degrade glycosaminoglycans (GAGs) in the lysosome. Lysosomal enzymes are crucial for the stepwise degradation of GAGs to provide a normal function of tissues and extracellular matrix. The deficiency of one or more lysosomal enzymes results in the accumulation of undegraded GAGs, causing cell, tissue, and organ dysfunction [1-3]. There are seven types of MPS that are categorized based on lack or defect in one of eleven specific lysosomal enzymes and are described as MPS I through MPS IX (excluding MPS V and VIII, which are no longer used) [1-3]. All MPS are autosomal recessive disorders except for MPS II, which is X-linked. Clinical features and severity vary accordingly to the type of MPS [1-3].

All MPSs are progressive in nature, and, without treatment, the severity of involvement increases with age [1-4]. Haemopoietic stem cell transplantation (HSCT) and enzyme replacement therapy (ERT) are two therapeutic strategies that can promote the improvement of airway deformities by reducing soft tissue accumulation and associated obstructive sleep apnoea (OSA) and pulmonary complications [5-8]. However, therapeutic options do not appear to significantly improve pre-existing neurocognitive dysfunction, skeletal abnormalities, or cardiac valvular dysfunction, especially if they are already advanced [5-8].

The global prevalence of all forms of MPS is estimated to be one in every 25,000 births [1]. However, MPS is underdiagnosed or misdiagnosed, especially in the milder forms of the disease, making it difficult to determine their true frequency in the general population. In Portugal, according to Khan et al., the incidence of MPS between 1981 and 2001 was 0.48 per 100,000 live births [1,2]. Regarding the age of diagnosis, the literature is not consensual. Some reports state that the age of first clinical signs in patients with a severe phenotype can be evidenced from birth, with referral to specialists as early as four months; other authors report diagnosis at five years, on average. However, in attenuated forms of the disease, diagnosis may be delayed until the age of 10-12 years [9].

As the disease progresses, involvement of various organs and systems occurs, requiring surgery to treat a variety of associated symptoms, including ear, throat, and nose problems, skeletal and articular malformations, hernias, hydrocephalus, and heart valve disorders [2,3,6]. Comprehensive data on the perioperative care of children with MPS is lacking, with sparse data consisting of only case reports and small case series [5]. There is a higher incidence of perioperative morbidity and mortality in this specific group of patients when compared to the general paediatric anaesthetic population [5,10-12]. For this reason, reviews of the anaesthetic management at reference centres are extremely valuable.

In view of this, we retrospectively reviewed the perioperative care, anaesthetic management, and complications of children with MPS cared for by our anaesthetic team over the past 12 years. We aimed to primarily focus on airway management and analyse the occurrence of intra and post-operative complications. We also included a comprehensive review of the literature.

## Materials and methods

After approval by the local health research committee, a descriptive and retrospective study was conducted in compliance with the National Ethics Committee regulations. Written informed consent was waived due to the retrospective design. Electronic medical records (including anaesthetic charts, diagnostic tests, and physician and nursing notes) of the 39 procedures in MPS patients under 18 years of age submitted for anaesthetic procedures between 2010 and 2021 at Centro Hospitalar Universitário São João, Oporto, Portugal, were assessed. Patients with incomplete data charts were excluded. Twenty-nine medical records of 10 patients fulfilled the eligibility criteria.

Demographic data, MPS type, organ involvement at the time of the procedure, American Society of Anesthesiologists (ASA) Physical Status classification, and procedure data were recorded. Anaesthetic approach, including induction technique, method of airway management used, number of attempts, extubation aspects, and complications in airway management were obtained from the anaesthetic chart. Information about post-operative management and associated complications were also collected. Data concerning this information were analysed using IBM® SPSS Statistics® 26 and were described in absolute and relative frequencies, and measures of mean and standard deviation (SD).

## Results

Demographic characteristics

Ten patients with a total number of 29 anaesthetic procedures were included. Five patients were males. Concerning the type of MPS, two patients presented with type I or Hurler phenotype, one with type II or Hunter phenotype, one with type IIIC or Sanfilippo, one with type IVA, one with type IB, three with type VI or Maroteaux-Lamy, and one with mucolipidosis type I, as summarized in Table 1.

**Table 1:** Demographic characteristics of patients of the study. MPS: mucopolysaccharidoses.

Demographic characteristics		
Number of patients, n		10
Gender, n (%)	Male	5 (50)
	Female	5 (50)
Type of MPS/mucolipidoses, n (%)	IH (Hurler)	2 (20)
	II (Hunter)	1 (10)
	IIIC (Sanfilippo)	1 (10)
	IV A	1 (10)
	IV B	1 (10)
	VI (Maroteaux-Lamy)	3 (30)
	Mucolipidosis type 1	1 (10)

Each patient was submitted to 2.9 procedures on average (standard deviation [SD] of 2.09). The lower age at the first procedure was two years old (y.o.), with 37.9% of the procedures performed between seven and 12 y.o. and 37.9% between 13 and 18 years (mean age of 8.8 y.o., SD 4.3). The majority of patients were ASA III (22 cases), and the information regarding the remaining ones is in Table 2. Regarding the past medical history and organ involvement, six patients presented with a history of cognitive impairment (two type IH, one type IIIC, two type VI, and one mucolipidosis type I), six with recurrent upper respiratory tract infections (tonsillitis, otitis) (one type IH, one type IIIC, one type IVB, two type VI, and one mucolipidosis type I), two with documented infiltration or oedema of the pharyngeal/laryngeal structures (1 type II and 1 type VI), and four with severe OSA with home non-invasive ventilation (one type IH, one type II, one type IVA, and one type VI). Regarding the cardiovascular system, seven patients had mild to moderate mitral regurgitation (one type IH, one type II, one type IIIC, one type IVB, two type VI, and one mucolipidosis type I) and one patient with MPS type VI had severe insufficiency already submitted to surgical repair by the time of the third anaesthetic procedure. Hypertension was reported in two patients with a diagnosis of MPS VI. One patient with MPS IH had undergone surgical correction of atrial communication, and another one with MPS type IVA presented with a ventricular sept defect. Cervical canal stenosis with medular compression was registered in four patients (one type II and three type VI), thoracolumbar scoliosis in five (one type II, one type IVB, two type VI, and one mucolipidosis type I), and deformities of the hip joint, limbs (valgus knee), and thorax (pectus carinatum) in five patients (one type IH, one type IIIC, one type IVB, and two type VI). Three patients presented with microcytic anaemia (IH and IVA) and two children with MPS type IH with thrombocytopenia. In terms of metabolic impairment, one patient with type IVB presented with type I diabetes mellitus and one with mucolipidosis type I with hypothyroidism. Table 3 summarizes the information regarding organ involvement according to the patient’s phenotype.

**Table 2:** Characteristics of the procedures included. SD: standard deviation.

Characterization of the procedure		
Number of procedures, n		29
Number of procedures in the same patient, mean (SD)		2.9 (2.09)
Age group, years, n (%)	1 to 3	2 (6.8)
	4 to 6	5 (17.1)
	7 to 12	11 (37.9)
	13 to 18	11 (37.9)
Mean age, years (SD)		8.8 (4.3)
Minimal age at the procedure, years		2
ASA classification, n (%)	II	7 (24.1)
	III	20 (69.0)
	IV	2 (6.9)
Enzymatic replacement at the time of the procedure, n (%)		19 (65.5)
Bone marrow transplant by the time of the procedure, n (%)		7 (24.1)
Surgical procedure, n (%)		26 (89.7)
Nonoperating room procedure, n (%)		3 (10.3)
Elective procedure, n (%)		29 (100)
Surgical specialty,n (%)	Orthopedics	12 (41.4)
	Otorhinolaryngology	6 (20.7)
	Paediatric surgery	4 (13.8)
	Neurosurgery	2 (6.9)
	Stomatology	1 (3.4)
	Paediatric surgery and orthopedics	1 (3.4)

**Table 3:** Absolute frequency of organ involvement accordingly patient’s phenotype. MPS: mucopolysaccharidoses; OSA: obstructive sleep apnoea; NIV: non-invasive ventilation.

Organ involvement accordingly patient’s phenotype								
		MPS IH (n=2)	MPS II (n=1)	MPS IIIC (n=1)	MPS IVA (n=1)	MPS IVB (n=1)	MPS VI (n=3)	Mucolipidoses type I (n=1)
Neurological system, n	Cognitive impairment	2		1			2	1
	Cervical canal stenosis with medullar compression		1				3	
Respiratory system, n	Infiltration/oedema of pharyngeal/laryngeal structures		1				1	
	Upper respiratory tract infections (tonsillitis, otitis)	1		1		1	2	1
	Severe OSA with NIV	1	1		1		1	
Cardiovascular system, n	Mild to moderate mitral insufficiency	1	1	1		1	2	1
	Surgical repaired atrial communication	1						
	Ventricular septal defect				1			
	Hypertension						2	
Bone and limbs, n	Thoracolumbar scoliosis	1				1	2	1
	Deformities of hip joint, valgus knee and pectus carinatum	1		1		1	2	
Haematological system, n	Microcytic anaemia	1			1			
	Thrombocytopenia	2						
Endocrine system, n	Type I diabetes mellitus					1		
	Hypothyroidism							1

Procedure characterization

Included procedures are presented in Table 2 and were all elective. Twenty-six cases were surgical interventions, and the remaining three were non-operating room procedures (magnetic resonance imaging (MRI), auditory evoked potentials, and echography). Regarding surgical interventions, 41.4% were orthopaedic, followed by 20.7% performed by otorhinolaryngology, 13.8% by paediatric surgery, 6.9% by neurosurgery, 3.4% by stomatology, and 3.4% in a multidisciplinary approach conducted by paediatric surgery and orthopaedics.

Pre-operative period

All cases had a pre-operative blood count and biochemistry analysis. Regarding cardiac evaluation, pre-operative electrocardiography was performed in 20 cases and echocardiography in 15. Pulmonary function tests were performed in four cases: one with documented restrictive ventilatory alteration, one with severe scoliosis, one with a history of recurrent respiratory tract infections, and the remainder with mild OSA. Pre-operative polysomnography was performed in two patients with known OSA. A computed tomography scan of the airway with the creation of three-dimensional reconstructions of the trachea and flexible endoscopy was performed on two patients.

Intra-operative period 

As represented in Table 4, general anaesthesia was performed in 19 cases (65.5%), combined anaesthesia (general and caudal epidural block or peripheral nerve block) in seven cases (24.1%), and spinal anaesthesia in three (10.3%).

**Table 4:** Description of the types of anaesthesia.

Type of anaesthesia		n (%)
General anaesthesia	Balanced general anaesthesia	14 (48.2)
	Inhalation anaesthesia	3 (10.3)
	Total intravenous anaesthesia	2 (6.9)
Combined anaesthesia	General anaesthesia and peripheral nerve block(s)	1 (3.4)
	General anaesthesia and caudal epidural block	6 (19.6)
Spinal anaesthesia		3 (10.3)

The intra-operative period’s main anaesthetic aspects are presented in Table 5. No cases of difficult bag-mask ventilation were registered. Airway management was achieved most commonly with endotracheal intubation (10 cases, 38.5%), followed by supraglottic airway with laryngeal mask airway (LMA) device in nine cases (34.6%), facial mask in five cases (19.2%), and intubation by established tracheostomy in two cases (7.7%). Regarding those submitted to endotracheal intubation, one case (mucolipidosis type I) was intubated before attending the operating room (OR), at the paediatric intensive care unit (PICU). In this case, intubation was performed with a fiberoptic bronchoscope (FOB) on the third attempt. Regarding endotracheal intubations performed in the OR, seven were performed initially with direct laryngoscopy with a Macintosh blade of paediatric size by the anaesthesiologist scheduled in that room. All three of them were successfully performed on their first attempt. Regarding failed first attempt cases, in two cases, both with a diagnosis of MPS type II, two failed attempts with direct laryngoscopy with a Macintosh blade were registered, followed by a third successful attempt with FOB. Information regarding the Cormack-Lehane grade of direct laryngoscopic views was not registered. In one case with MPS type IH, a second attempt was performed with a paediatric C-MAC® video laryngoscope (VLG), with success on the third attempt with that device. In one case involving MPS type VI, intubation was accomplished on the second attempt with the help of an introducer. FOB was the first option in one patient successfully intubated at the first attempt under inhalational anaesthesia with spontaneous breathing. This patient was pre-operatively submitted to flexible endoscopy describing arytenoid oedema. Information regarding fiberscope was not registered.

**Table 5:** Intra-operative anaesthetic patterns. FOB: fiberoptic bronchoscopy; LMA: Laryngeal mask airway; NBD: neuromuscular blocking drug; OR: operating room; PICU: paediatric intensive care unit; VLG: video laryngoscopy

Intraoperative anaesthetic patterns				n
Airway technique	Facial mask			5
	LMA			9
	Naso/Orotracheal tube			10
	Tracheostomy			2
First attempt airway approach at the OR	Direct laryngoscopy with Macintosh	Successful on first attempt		3
		Failed first attempt (n=3)	VLG on third attempt	1
			FOB on third attempt	2
		Missing information		1
	VLG	Successful on second attempt		1
	FOB	Successful on firstattempt		1
Use of neuromuscular blocking drugs on airway management				3
Number of anaesthesiologists in OR	1			24
	2			2
	Missing data			3
Presence of other specialties in OR	Pulmonologist			4
	Otolaryngologist			6
Induction technique	Inhalational with sevoflurane			14
	Intravenous	Propofol		7
		Propofol and midazolam		1
		Ketamin and midazolam		1
	Missing information			3
Ventilation	Spontaneous			14
	Controlled			10
	Assisted			1
	Missing			1
Airway complications	At extubation in OR		Bronchospasm	1
	At extubation in PICU		Stridor	2
Management of extubation	Extubation in OR			6
	Tracheostomy in OR			1
	Extubation in PICU (first 24 hours)			3

In general or combined anaesthetic techniques, inhalation induction with spontaneous breathing was the preferred induction technique (14 cases, 53.8%), followed by intravenous induction (nine cases, 34.6%). The most frequent intravenous drugs used were fentanyl and propofol (seven cases). In three cases, neuromuscular blockade was performed before intubation: one with rocuronium and two with suxamethonium. There was no record of airway complications on intubation, except for one concerning difficult intubation in five cases.

Extubation was performed in the OR with no complications in six cases. One patient was electively tracheostomized at the OR by the end of the procedure. No information concerning the use of airway exchange catheters or extubation for non-invasive ventilation was registered. In one tracheotomized patient, bronchospasm was registered in the anaesthetic emergency. In three cases, the patient was transferred to the PICU intubated.

In terms of staff, two anaesthesiologists were scheduled in the OR in two cases. A paediatric pulmonologist was present in four cases and performed intubation by fibroscopy in three cases. The presence of an otolaryngologist was verified in the six cases of otolaryngology surgery.

Post-operative period

Immediate post-operative care was provided in the post-anaesthesia care unit (PACU) in 21 procedures (72.4%) and in the PICU in eight (27.6%). PICU admitted patients who were submitted for tonsillectomy, myringotomy, myringotomy and tracheostomy, femoral and tarsal osteotomy, posterior cervical decompression (two patients), and posterior column fixation. Three of them were admitted under invasive mechanical ventilation after cervical decompression, posterior column fixation, and tonsillectomy. The last one remained intubated due to difficult intubation. Extubation of all these patients was performed during the first 24 hours. At the time of extubation, two of them developed stridor and one needed reintubation.

In what concerns to post-operative complications, nausea and vomiting were registered in three cases (10.3%), pulmonary complications in five cases (17.2%) (two cases of stridor after extubation, transient desaturation, and pneumonia), nosocomial meningitis in one case (3.4%), and acute anaemia needing transfusion in another one (3.4%). No cardiovascular complications were reported. All complications occurred in different patients except for post-operative nausea and vomiting, in which two of them occurred in the same patient at different orthopaedic surgical interventions (one at 11 years old and one at 14 years old), despite the triple anti-emetic prophylaxis on the second surgery.

Regarding the length of PICU stay, four patients remained there up to 48 hours, two between 48 and 72 hours, and two for a week. No deaths were registered in the first 30 days after the procedure.

At the time of the study, two patients had died: one at the age of 16 and the other one at 13 years of age. Table 6 summarizes the post-operative data.

**Table 6:** Post-operative data. PACU: post-anaesthesia care unit; PICU: paediatric intensive care unit.

Post-operative data		n
Post-operative respiratory complications	Stridor	2
	Desaturation	2
	Pulmonary infection	1
Post-operative vigilance location	PACU	20
	PICU	8
	Missing information	1
Length of PICU stay, hours	24–48	4
	49–72	2
	>72	2

## Discussion

GAG accumulation in the airway, lungs, heart, and musculoskeletal system provides a double concern to the anaesthetic provider [3,7,8,13]. Firstly, organ dysfunction resulting from GAG accumulation predisposes MPS patients to multiple surgical interventions [3,6,9,13]. In this study, each patient underwent an average of 2.9 surgical procedures. However, that number is probably greater due to undocumented interventions before 2010. Secondly, GAGs deposit and related organ dysfunction increase the anaesthetic risk [3,7,13]. GAGs accumulation in the upper airway leads to hypertrophy of the adenoids, tonsils, tongue, and laryngopharynx, which can predispose to a difficult airway [4,7,14,15]. Besides that, these patients can also have a higher-positioned epiglottis and limited mouth opening secondary to temporomandibular joint dysplasia and micrognathia [15]. Some patients, especially those with MPS IVA [8], can also have tortuous and narrowed tracheas and tracheomalacia [3,5,7,14-17]. Based on this evidence, a computed tomography scan of the airway with the creation of three-dimensional reconstructions of the trachea and flexible endoscopy is desirable in these patients [3,5,7,14,15]. Skeletal dysplasia such as atlantoaxial instability, spinal cord compression mainly at the craniocervical and thoracolumbar regions, limited neck mobility, pectus carinatum, and scoliosis are features frequently found in these patients which can potentiate a difficult airway [3,5,7,10,12]. Atlantoaxial instability due to odontoid hypoplasia leads to an unstable cervical spine [11,16]. Neck movement during intubation can lead to subluxation at the atlantoaxial region causing spinal cord damage and paralysis [16]. Care must be taken at the time of induction, positioning, and emergence to maintain the head and neck at a neutral position with manual in-line stabilization during these critical periods [7]. Spinal compression can also occur during long procedures or procedures requiring head movement. Therefore, besides a careful neurological examination with assessment of hyperreflexia, a magnetic resonance imaging scan of the spine in a neutral position and a flexion/extension X-ray of the spine can be recommended before the procedure to assess the risk of spinal cord compression [3,15,17]. Intra-operative somatosensory evoked potential monitoring is recommended during long procedures to exclude any complications related to spinal cord compression [3,17].

As previously mentioned, a large number of patients with MPS will need surgical intervention. Regarding age, cooperation and tolerance of the child, and the type of procedures, the majority will be carried out under general anaesthesia [8]. This implies towards the management of a potentially difficult airway. Attending to our sample, approximately 90% of procedures were performed under general anaesthesia. In the majority of procedures, induction was performed with sevoflurane, ensuring spontaneous ventilation. There was no report of difficult bag-mask ventilation despite the multiple factors that can predispose to it. Although there is no description of the position in which induction was performed, multiple articles report that placing the patient in a lateral position can help to maintain the airway patency during the induction phase, as in the supine position, gravity leads the tongue to obstruct the airway in patients with OSA [6,12,13,17]. Face mask or LMA was chosen in 53.8% of cases, reducing the incidence of airway manipulation-related complications [6,12,13,17]. Nonetheless, it is important to be aware that spontaneous breathing with a face mask can be challenging due to anatomical issues pertaining to airways or excessive secretions, as well as LMA insertion due to reduced mouth opening and a large tongue [9]. Tracheal intubation problems with conventional laryngoscopes frequently occur in MPS patients due to thickened and stiffened tissues in the laryngopharynx and trachea [3,5,9]. In our study, 50% of direct laryngoscopy intubation attempts with a Macintosh blade failed on the first attempt, highlighting the advantage of transitioning to indirect techniques at the first attempt. We believe that this high percentage of direct laryngoscopy can be related to the greater number of interventions that were performed in a period when indirect methods were not available and used widely in anaesthetic practice. As institutional experience and availability of sophisticated airway instruments vary broadly, there is not a specific type of indirect intubation technique that can be recommended in children with MPS [12-17]. The literature describes favourable results either with VLG or FOB [3,5,8,10,12]. Some authors highlight that FOB through a supraglottic device in children with MPS could be the preferential technique as it allows the recognition of subglottic or tracheal pathology that was not previously documented and the possibility to remain head and cervical aligned without cervical extension in the presence of cervical spinal canal stenosis or atlantoaxial instability [5,9,12,13]. Others, however, attending to the more widespread availability of VLG, defend that this technique may become a preferred technique for endotracheal intubation in MPS patients [10]. Hack et al. and Walker and Garbarino suggest the potential disadvantages of using VLG in these patients related to the absence of awareness of subglottic or tracheal stenosis and the potential for trauma and bleeding even with the most carefully placed laryngoscope blade, rendering direct oxygenation and/or anaesthesia impossible [5,7].

Anaesthetic issues extend to the lower respiratory tract. The respiratory function of MPS patients can be highly disproportional to the patient’s clinical appearance. These patients frequently have restrictive or obstructive lung disease, due to scoliosis and thoracic cage abnormalities and deposits on alveolar and interstitial pulmonary spaces, recurrent upper and lung infections, and OSA which can lead to difficult ventilation, desaturation and bronchospasm at induction and emergence [3,5,7,13,14,17]. Regarding OSA, its presence and severity is a good indicator of the severity of airway problems and airway complications during anaesthesia. Based on this, literature recommends a sleep study or polysomnography before any anaesthetic procedure in every patient that has a suspicion of OSA based on clinical evaluation [3,17]. Clinical evaluation is performed by the application of questionnaires suitable for the paediatric population to address OSA symptoms, like the Paediatric Sleep Questionnaire, I’M SLEEPY questionnaire, or the paediatric version of the Epworth Sleepiness Scale; physical examination focused on craniofacial and ear, nose, and throat abnormalities; and eventually an endoscopic assessment to evaluate the patency of the nasal cavities [3,17,18]. Attending to our sample, only four patients were diagnosed with OSA, with pre-operative polysomnography performed on two of them at the time of the study. Unlike other patients in whom respiratory function tests like spirometry are extremely valuable in measuring respiratory function, in MPS patients the results are difficult to interpret due to lack of reference data and cooperation problems, which lead to erroneous results [7]. Based on the literature, pre-operative optimization could involve an evaluation by a pulmonologist, with the help of physiotherapists and nutritionists, to optimise respiratory function as well as treatment of underlying chronic infection and OSA [7], with home continuous or bi-level positive pressure as being found in three of four patients with severe OSA.

Although all patients had cardiac involvement, no perioperative cardiovascular events were noted in our study. According to the literature, cardiac valvular disease, with mitral and aortic regurgitation, is the most common finding in MPS patients. Progressive thickening due to GAG deposition causes either mitral or aortic stenosis and/or regurgitation [3,7,19,20]. These patients can also present with complete atrioventricular block [3,7,19]. Evaluation of cardiac risk prior to general anaesthesia is crucial, including assessment of heart function, detecting those with increased risk of myocardial ischemia and conduction abnormalities, and providing recommendations for additional medications or tests, level of post-operative care, and the need to defer the procedure [20]. Regarding heart function, an echocardiogram, which was performed in 51.7% of our cases, with colour flow and Doppler of cardiac valves should be performed routinely in MPS to determine the severity of valve disease [20]. Concerning myocardial ischemia, identifying MPS patients who may be at increased risk during anaesthesia can be very difficult as clinical signs may be masked pre-operatively due to patient physical inactivity and communication difficulties [20]. For that, a dobutamine stress echocardiogram, not routinely implemented in MPS patients, could be a good option for unmasking underperfused areas of myocardium attending to its safety and non-invasiveness. Symptoms of syncope or presyncope and evidence of any potential rhythm disturbance on the ECG should always be evaluated. The presence of a PR interval at the upper limits of normal for age merits further investigation, such as Holter monitoring. For patients with suspected cardiomyopathy, the ejection fraction and B-type natriuretic peptide measurements are important as baseline values and as a means of assessing response to therapy [20].

Regarding the difficulty of intubation, it is equally important to formulate an extubation plan [3]. That can be achieved with tube changers, allowing reintubation when concerns exist about successful extubation. Despite this, we did not find evidence of advanced extubation techniques performed in our institution in these cases.

Another important aspect is the therapeutic options that are available to these patients. Recombinant human enzyme as weekly venous infusions is now available for MPS I, II, IV, and VI, and evidence shows that although the response is variable, ERT often produces significant improvement in the altered airway, by reducing soft tissue accumulation and, therefore, OSA and pulmonary complications, together with a reduction in hepatosplenomegaly, improves cosmetic and health-related quality of life (HRQoL) parameters [5-8]. HRQoL could be evaluated by addressing activities of daily living (ADL) as mobility, self-care, independence in usual activities like personal hygiene and dressing, pain and/or discomfort, anxiety, depression, energy, sleep quality, conversation, self-esteem, and school status [21]. ADL could be evaluated before ERT implementation by applying questionnaires such as EuroQol 5D and Pediatric Quality of Life (PedsQL) and after it using TNO-AZL Child Quality of Life (TACQOL) and TNO-AZL Preschool Children’s Quality of Life (TAPQOL) questionnaires, which are specific for a certain type of MPS [21]. As with ERT, haemopoietic stem cell transplantation reduces GAG deposits on soft tissue, improving the airway issues of MPS patients. However, it presents the limited capability to reduce skeletal, cardiac valvular, or neurocognitive dysfunction, especially when already well-established [5-7].

At the time of each procedure, 65.5% of patients were under ERT, which can explain the low number of adverse airway events. Nevertheless, the small sample number and incomplete data in our study suggest the need for more quality data on the matter, which might reinforce these findings elsewhere. Regarding valvular dysfunction, neurological and skeletal deformities, ERT does not appear to have the same favourable results due to the enzyme’s inability to cross the blood-brain barrier and the valvular and skeletal tissues’ relatively poor blood supply [5].

Regarding post-operative surveillance, eight patients were admitted to the PICU, including patients submitted for tonsillectomy, myringotomy, and femoral and tarsal osteotomies. This highlights the need for a high level of care in these patients, attending to intra-operative complications related to organ dysfunction and airway management problems.

To maximally optimize these patients pre-operatively, define a structured plan for airway management and minimize the risk of intra and post-operative complications, our hospital, which is a reference centre for MPS patients since 2015, developed a multidisciplinary MPS team, which involves anaesthesiology since 2002, paediatric pulmonology, paediatric cardiology, and surgical specialties according to the procedure. A team briefing occurs anytime an MPS patient is proposed for surgery and allows discussion of specific clinical cases of patients awaiting surgery and the definition of perioperative strategies and plans according to the latest evidence in this area. Beyond this, the presence of two experienced anaesthesiologists at the OR is important. The presence of otolaryngology should be considered in specific cases, particularly when front-of-neck access or management is in mind. In our study, only two cases were reported with the presence of two anaesthesiologists, and only four cases were counted with paediatric pulmonology who performed intubation by FOB in three of them. The presence of otorhinolaryngology only occurred in the six cases of surgery of this specialty.

Our study has several limitations. First, its retrospective nature and its potential source of bias. Second, our data only reflect the clinical experience of one Portuguese centre with a smaller sample due to the rarity of the disease. Due to the reduced sample size, we were unable to perform statistical analyses regarding perioperative adverse events, mainly airway and respiratory events, in that population and correlate those events with patient variables, namely age or MPS type. According to literature, the incidence of airway and respiratory events decreases with age in patients who are subjected to therapeutic effects of instituted treatment, which may slow down disease progression [6,7].

## Conclusions

Patients with MPS represent a huge challenge for the anaesthesiologist. Elective surgery requires an extended pre-operative evaluation of anaesthetic risk factors. These patients pose difficulties during intubation, including the potential for airway critical events, such as desaturation, stridor, and bronchospasm, demanding a transition to indirect methods of airway management. Literature remains inconclusive regarding the best approach to airway management, although FOB through LMA is gaining popularity in the past few years. Also, it is important to consider that the spectrum of airway management equipment available in many institutions is widening. An anaesthetic plan should be laid down by an experienced team for MPS patients. A prospective study would be useful to assure a higher quality of clinical records, allowing a more accurate description and analyses of perioperative aspects of MPS patients’ management.
